# Safety of 100 µg venom immunotherapy rush protocols in children compared to adults

**DOI:** 10.1186/s13223-017-0204-y

**Published:** 2017-07-12

**Authors:** Johanna Stoevesandt, Christine Hosp, Andreas Kerstan, Axel Trautmann

**Affiliations:** Department of Dermatology, Venereology, and Allergology, University Hospital Würzburg, Josef-Schneider-Straße 2, 97080 Würzburg, Germany

**Keywords:** Anaphylaxis, Bee, Buildup phase, *Hymenoptera*, Pediatric, Risk factor, *Vespula*

## Abstract

**Background:**

There is a paucity of studies examining the safety of venom immunotherapy (VIT) in children. We aimed to assess the incidence of anaphylactic side effects during rush VIT in a cohort of pediatric patients and adult controls.

**Methods:**

72 consecutive cycles of VIT-buildup in 71 children/adolescents aged 7–17 years were retrospectively evaluated and compared to an adult control group (n = 981) with regard to baseline parameters (sex, causative venom, severity of index sting reaction, results of allergy testing, comorbidities) and the incidence of anaphylactic adverse reactions.

**Results:**

Compared to adults, severe index sting-induced anaphylaxis was significantly less common in children (*P* = .001). Children were more likely to suffer from bee venom allergy (*P* < .001) and showed higher levels of bee venom-specific IgE (*P* = .013), but lower serum tryptase concentrations (*P* = .014). The overall rate of VIT-induced anaphylactic reactions was higher in children than in adults (6.9% vs 2.5%, *P* = .046 by univariate analysis). In the final binary logistic regression model, however, only bee VIT (*P* = .039; odds ratio 2.25; confidence interval 1.04–4.87) and 5-day compared to 3-day buildup protocols (*P* = .011; odds ratio 2.64; confidence interval 1.25–5.57) were associated with an increased risk of treatment-induced anaphylaxis. All pediatric patients finally reached and tolerated the target maintenance dose of 100 µg.

**Conclusions:**

The higher anaphylactic reaction rate observed in pediatric patients may be attributed to a greater prevalence of bee venom allergy. VIT-induced anaphylaxis in children is usually mild and does not affect further updosing and maintenance of VIT.

## Background

As a result of age-specific outdoor activities, children are frequently exposed to stings by *Vespula* species or honey bees. Nonetheless, sting-induced anaphylaxis is considered to be infrequent. Epidemiologic studies dating from the early 70s and late 90s reported *Hymenoptera* sting-induced pediatric anaphylactic reaction rates to be as low as .3–.4% [1, 2]. A more recent questionnaire-based study in Irish school children, however, described a prevalence of 1.5%, which is closer to data derived from adult populations [3–5].

Sting-induced anaphylaxis commonly takes a benign course in pediatric patients, meaning that, first, reactions tend to be less severe than in adults [6–8] and, second, anaphylaxis is less likely to recur in the case of future re-stings [9]. Untreated children who have experienced moderate to severe index sting reactions, however, are at an increased risk of relapse compared to those with a history of urticaria or angioedema only, and the severity of recurring anaphylaxis was found to correspond to that of the initial reaction [10, 11]. Based on these observations, international guidelines state that venom immunotherapy (VIT) should be recommended for children with a history of moderate to severe sting-induced anaphylaxis, but not for those with only urticaria and/or angioedema [4, 5].

Several studies confirm the effectiveness of VIT in pediatric patients [9, 10, 12–14], and children are commonly considered to tolerate treatment at least as well or even better than adults [8]. There are, however, a surprisingly small number of studies addressing the safety of VIT in pediatric patients [12, 15–18], and even fewer including a direct comparison to adults [17, 18]. We aimed to assess the inpatient buildup phase of VIT using 3- or 5-day rush protocols in a cohort of pediatric patients, to examine the incidence of VIT-induced adverse reactions compared to a large and homogeneous adult control group and to work out age-specific characteristics.

## Methods

### Patients

Medical records of 1052 consecutive patients who had received at least one cycle of inpatient VIT buildup between January 2004 and May 2016 were available for retrospective evaluation. All patient-related procedures were part of routine practice; written informed consent was obtained from the patients and/or the patients’ caregivers for allergological work-up and initiation of VIT.

### Collection of data

Clinical data and the results of allergy testing were retrieved from the medical records. Information on VIT-induced adverse reactions was gathered from standardized inpatient treatment protocols. In patients receiving more than one cycle of VIT buildup due to either double allergy to bee and *Vespula* venom, or following discontinuation of VIT for various reasons, each treatment cycle was evaluated separately. The local institutional review board consented to the retrospective review and publication of anonymized clinical data. Data from overlapping patient cohorts were part of previous publications [19, 20]; 309 patients (291 adults; 18 children/adolescents) were added for the current evaluation.

### Allergy testing

Intradermal skin testing with *Hymenoptera* venoms (ALK-Abelló, Wedel, Germany) was carried out according to international guidelines [21]. Venom-specific serum IgE was measured using the ImmunoCAP™ method (Thermo Fisher Scientific, Freiburg, Germany). Baseline serum tryptase concentrations were determined using ImmunoCAP Tryptase™ (Thermo Fisher Scientific) in a subgroup of 628 patients including all patients with a history of severe sting-induced anaphylaxis.

### Venom immunotherapy

VIT was indicated for adults with systemic sting reactions of any severity, though it was considered optional in the case of mild reactions only. Treatment was recommended for children with moderate to severe anaphylaxis, but not for those with urticaria/angioedema as the only symptom (exceptions were made for individual reasons such as high risk of exposure or impairment of quality of life). Aqueous allergen solutions (ALK-lyophilisiert SQ™, ALK-Abelló) were used for VIT buildup. The venom maintenance dose of 100 µg was achieved using standardized 3- and 5-day inpatient buildup protocols comprising 9 injections and a total dose of 301.1 µg, or 14 injections and a cumulative dose of 311.5 µg respectively (Table 1). 5-day protocols were used for all buildup-cycles before 2007, 3-day protocols were used afterwards. All injections were administered by trained physicians. Preventive medication with antihistamines was not routinely given.

**Table 1:** Three and 5-day inpatient rush protocols for VIT buildup

Protocol	3 days		5 days	
	Day	Dose (µg)	Day	Dose (µg)
	1	.1	1	.02
		1		.08
		10		.2
				.4
	2	20	2	.8
		30		2
		40		4
				6
	3	50	3	8
		50		20
				40
	4 or 5^a^	100^b^	4	50
				80
			5	100^b^
Cumulative dose (µg)		301.1		311.5
Number of injections (n)		9		14

^a^Outpatient follow-up injection

^b^Aluminium hydroxide adsorbed depot preparation

### Classification of VIT-related adverse reactions and grading of anaphylaxis

VIT-related systemic reactions were classified as “objective” if any of the following symptoms occurred: urticaria with or without angioedema, respiratory compromise as a result of bronchospasm or laryngeal edema, arterial hypotension or collapse, distinct gastrointestinal involvement such as abdominal cramps, diarrhea, or vomiting [22]. Any undefined symptoms including sensations of warmth, pruritus, or dizziness were categorized as “subjective” as long as they were not accompanied or followed by the above defined objective signs. The severity of anaphylaxis was graded as mild (grade I), moderate (grade II), or severe (grade III) according to the classification system proposed by Muraro et al. [23].

### Statistical analysis

Statistical analyses were performed with SPSS version 23 for Windows (SPSS Inc., Chicago, Ill). Interval-scaled data are reported as median and interquartile range (IQR). Ordinally and categorically scaled data are presented as absolute and relative frequencies. The Mann–Whitney U test was done to compare two groups for interval-scaled data. The Chi square or, if applicable, Fisher’s exact test were conducted for ordinal and categorical data. A binary logistic regression model was used to estimate the probability of a VIT-induced anaphylactic reaction in relation to several predictor variables (age group, venom, injection protocol). All tests were 2-tailed, and *P* values <.05 were considered statistically significant.

## Results

### Patient cohort

**Fig. 1:**
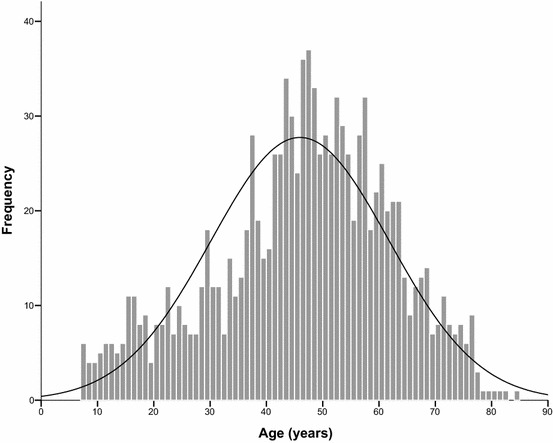
Age distribution of the total group

Figure 1 provides an overview on the total group’s age distribution. Of the total cohort (n = 1052), 71 (6.7%) were children or adolescents aged 7–17 years. Pediatric patients differed from adults with regard to a number of clinical baseline parameters (Table 2). A history of severe (grade III) index sting reactions was documented in only 9.9% of cases compared to 26.5% in adults (*P* = .001). There was a significantly higher proportion of bee venom allergic individuals among pediatric patients (32.4% vs 14.7%, *P* < .001). Bee venom allergic children had a significantly higher specific IgE than adults (median 15.3 kU/L vs 8.0 kU/L, *P* = .013), whereas the concentrations of specific IgE in children and adults sensitized to *Vespula* venom did not significantly differ (median 6.7 kU/L vs 4.8 kU/L, *P* = .37). Lower baseline serum tryptase concentrations were found in children (median 3.5 µg/L vs 4.4 µg/L, *P* = .014). No cardiovascular comorbidities were found in pediatric patients (0% vs 29.5% in adults, *P* < .001), and no children were on cardiovascular medication as opposed to 277 (28.2%) adults (*P* < .001). No significant differences were found with regard to the sex ratio (*P* = .17), the results of intradermal testing (*P* = .15), the latency time between the index sting and the initiation of VIT (*P* = .19), and/or the use of 3 or 5-day dose increase protocols (*P* = .53).

**Table 2:** Clinical baseline parameters of patient cohort

	Total		Children		Adults		*P* value^a^
	n	%	n	%	n	%	
Age (years) at VIT-initiation, median (IQR)	47 (20)		14 (6)		48 (19)		
Sex							
Male	580	55.1	45	63.4	535	54.5	.17
Female	472	44.9	26	36.6	446	45.5	
Diagnosis: IgE-mediated allergy to							
Bee venom	167	15.9	23	32.4	144	14.7	<.001
*Vespula* venom	856	81.4	47	66.2	809	82.5	
Bee and *Vespula* venom	29	2.8	1	1.4	28	2.9	
IgE to causative insect (kU/L), median (IQR)							
Bee venom	8.7 (22.2)		15.3 (36.6)		8.0 (21.0)		.013
*Vespula* venom	4.8 (11.4)		6.7 (15.5)		4.8 (11.2)		.37
Baseline serum tryptase concentration							
Availability^b^	628	59.7	33	46.5	595	60.7	
Median (µg/L) (IQR)	4.4 (3.2)		3.5 (1.9)		4.4 (3.2)		.014
Severity of index sting-induced anaphylaxis							
Grade I (mild)	138	13.1	5	7.0	133	13.6	
Grade II (moderate)	647	61.5	59	83.1	588	59.9	
Grade III (severe)	267	25.4	7	9.9	260	26.5	.001
Cardiovascular comorbidities	289	27.5	0	0	289	29.5	<.001
Concurrent cardiovascular medication							
Any	277	26.3	0	0	277	28.2	<.001
ACE-inhibitor	131	12.5	0	0	131	13.4	<.001
Beta-blocker	59	5.6	0	0	59	6	.028
Latency (months) from index sting to VIT, median (IQR)	7 (7)		8 (6)		7 (7.5)		.19

*IQR* interquartile range

^a^Based on univariate analysis comparing children and adults

^b^Baseline tryptase concentrations were determined in a subgroup of 628 patients (59.7%) including all patients with a history of severe index sting-induced anaphylaxis

### Course of VIT

1101 consecutive cycles of VIT buildup were evaluated (children: 72 cycles; adults: 1029 cycles). 47 patients (1 child; 46 adults) underwent more than one cycle due to double allergy to honey bee and *Vespula* venom (1 child; 28 adults) or following discontinuation of VIT for various reasons (18 adults). In total, 728 injections were given to children, and 10,217 to adults.

**Fig. 2:**
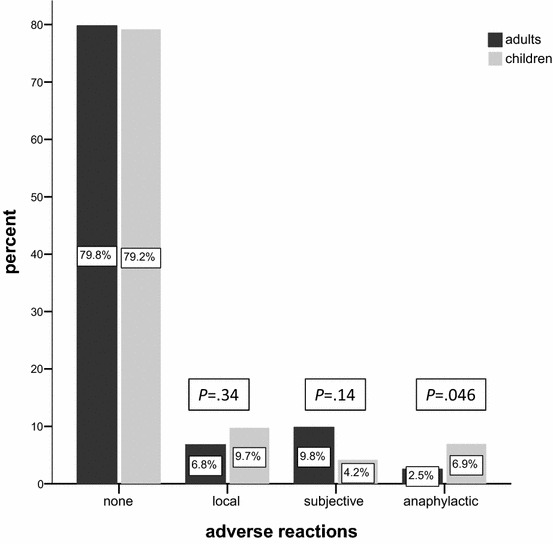
Large local reactions, subjective symptoms, and objective anaphylaxis during 1101 cycles of VIT buildup

**Fig. 3:**
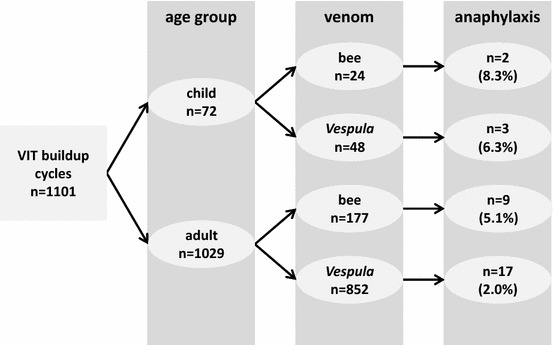
Incidence of VIT-induced anaphylactic reactions in venom-allergic children and adults

Figure 2 provides information on the course of VIT in both age groups. 878 (79.7%) cycles of VIT buildup were administered without any complications (children: 79.2%; adults: 79.8%). In the total cohort, there were 77 (7.0%) reports of large local reactions (children: 9.7%; adults: 6.8%, *P* = .34). 104 (9.4%) patients (children: 4.2%; adults: 9.8%, *P* = .14) complained of subjective symptoms which did not fulfill the above defined criteria of objective anaphylaxis. There were 31 (children: 5; adults: 26) reports of VIT-induced anaphylactic reactions. Respective objective reaction rates were 2.8% (total), 6.9% (children), and 2.5% (adults). The higher objective reaction rate observed in children was statistically significant in the preliminary univariate analysis (*P* = .046). The highest reaction rate (8.3%) was observed in the subgroup of bee venom allergic children (Fig. 3).

Anti-allergic treatment was given during 12 (16.7%) pediatric treatment cycles (oral antihistamine: 12.5%; intravenous steroid and/or antihistamine: 4.2%) and during 126 (12.2%) buildup cycles in adults (oral antihistamine: 10.2%; intravenous steroid and/or antihistamine: 2.0%). The difference was not statistically significant (*P* = .27).

### Predictors of VIT-induced anaphylaxis

Of several parameters (causative venom, injection protocol, sex, severity of index sting reaction, tryptase elevation), two (venom, protocol) were likewise associated with the incidence of VIT-induced objective anaphylaxis in a preliminary univariate analysis (data not shown). Honey bee VIT (*P* = .039; odds ratio 2.25; confidence interval 1.04–4.87) and 5-day compared to 3-day injection protocols (*P* = .011; odds ratio 2.64; confidence interval 1.25–5.57) were found to be associated with an increased incidence of anaphylactic adverse reactions in a multivariate binary regression model whereas childhood/adolescence was not confirmed as an independent risk factor (*P* = .10; odds ratio 2.35; confidence interval .85–6.52).

### Severity grading of VIT-induced anaphylaxis in pediatric patients

VIT-related anaphylaxis occurred in 5 children and adolescents aged 7–17 years (Table 3). 4 mild (grade I) reactions remained confined to the skin and responded well to treatment with antihistamines alone. In a 7-year old bee venom allergic girl, VIT-induced anaphylaxis was classified as moderate (grade II) due to additional bronchospasm. Her condition promptly improved upon treatment with inhalative salbutamol, intravenous antihistamines and corticosteroids. No epinephrine was given. The target venom maintenance dose of 100 µg was invariably reached and tolerated in all 5 reactors either within the regular inpatient dose increase schedule (n = 2) or following individually adjusted outpatient updosing protocols (n = 3).

**Table 3:** Clinical details on VIT-induced anaphylactic reactions in children and adolescents

Patient no.	1	2	3	4	5
History					
Age (years)	7	7	7	15	17
Sex	F	M	M	F	M
Causative venom	Bee	*Vespula*	*Vespula*	*Vespula*	Bee
Severity of anaphylaxis at index sting (grade)	II	II	II	III	II
Relevant comorbidities	None	None	Asthma	None	None
Concurrent medication	None	None	Yes^a^	None	None
VIT-induced anaphylaxis					
Severity (grade)	II	I	I	I	I
Symptoms					
Urticaria/angioedema	+	+	+	+	+
Respiratory	+	−	−	−	−
Gastrointestinal	+	−	−	−	−
Cardiovascular	−	−	−	−	−
Injection protocol	3 days	3 days	3 days	3 days	5 days
Venom dose prior to reaction (µg)	50	40	50	40	8
Time interval to injection (minutes)	15	60	120	25	60
Treatment					
Antihistamine	i.v.	Oral	i.v.	i.v.	Oral
Steroid	i.v.	−	−	−	−
Epinephrine	−	−	−	−	−
Inhalative beta2-adrenergic agonist	+	−	−	−	−

^a^Inhalative budesonide/formoterol

## Discussion

VIT effectively protects venom allergic children and adults from future sting-induced anaphylaxis [9, 10, 12–14, 24]. VIT itself, however, is a potential elicitor of anaphylactic side effects, which are most likely to occur during the dose increase phase [24–27]. As opposed to ample data derived from adult populations, there is a paucity of studies examining the safety of VIT in children [12, 15–18]. Our present study including a total of 1052 individuals, 71 of whom were pediatric patients, was designed to compare VIT-related adverse reaction rates in children and adults and to assess age-specific clinical parameters.

### Age-specific baseline characteristics of pediatric candidates for VIT

A preponderance of pediatric patients with a history of severe index sting-induced reactions might be expected to result from the fact that VIT was not recommended for children with mild (grade I) reactions only. Pediatric patients, however, reported a significantly lower rate of severe (grade III) index sting-related anaphylactic reactions than adults (9.9% vs 26.5%, *P* = .001). This is in accordance with previous publications stating that severe *Hymenoptera* sting-induced anaphylaxis is indeed very uncommon in children [6–9].

Our observation of a higher proportion of bee venom allergic subjects in the pediatric compared to the adult group likewise corroborates previously published data [7, 8, 18]. The majority of our young bee venom allergic patients (15 out of 23, i.e. 65.2%, data not shown) had a history of repetitive contact to bees due to beekeeping activities of family members or friends. We assume that high exposure is a risk factor for an early age onset of bee venom allergy. Our findings of higher bee venom-specific IgE levels in children (*P* = .013) and of an age-dependant increase of baseline serum tryptase concentrations (*P* = .014) are again in accordance with previous publications [18, 28]. It is tempting to speculate that lower serum tryptase concentrations during childhood might decrease the likelihood of severe anaphylactic sting reactions. Another important reason for the benign natural course of *Hymenoptera* venom allergy in children [6–9] might be the virtual absence of cardiovascular comorbidities which are considered an important risk factor for severe or even fatal anaphylaxis in adults [29].

### Incidence of VIT-induced anaphylaxis in children versus adults

The VIT-related anaphylactic reaction rate (2.5% referring to the total number of treatment cycles) observed in our adult group is below that of previous studies which did not require documentation of objective symptoms to define an anaphylactic adverse reaction [25–27]. In this context, we would like to stress the importance of an accurate definition of anaphylaxis as misinterpretation of anxiety-related subjective symptoms may lead to an over-estimation of anaphylaxis rates, particularly in adults. Accordingly, we found a tendency towards a higher incidence of subjective symptoms in our adult group which, however, did not reach statistical significance (*P* = .14).

The pediatric VIT-related anaphylactic reaction rate of 6.9% in our series fits well within the—remarkably broad—range of previously published data. It slightly exceeds the values reported by Konstaninou et al. and Nittner-Marszalska et al. (3.7% respectively) [12, 18]. The higher rates described by Köhli-Wiesner et al. (16%), Birnbaum et al. (10.8%), and, most recently, by Confino-Cohen et al. (19%) might be attributed to high proportions of bee venom allergic children [15–17].

Whereas the tendency towards a higher objective reaction rate in our pediatric group in relation to the adult controls was statistically significant in the preliminary univariate analysis (P = .046), only bee VIT (*P* = .039; odds ratio 2.25; confidence interval 1.04–4.87) and the use of the 5-day injection protocol (*P* = .011; odds ratio 2.64; confidence interval 1.25–5.57) were found to be associated with an increased risk of VIT-induced anaphylaxis in the final multivariate model. Young age alone was not confirmed as an independent risk factor (*P* = .10). The use of bee venom has long been identified as a major predictor of VIT-induced anaphylactic side effects [14, 16–18, 24–26, 30]. Inpatient dose increase protocols including a higher number of injections and/or greater cumulative doses have likewise been shown to be associated with an increased incidence of treatment-related anaphylaxis [19, 30]. Children and adults in our series, however, did not differ with regard to the protocols used for VIT-buildup (*P* = .53). We conclude that the higher rate of VIT-induced anaphylactic reactions observed in our pediatric group might result from the greater proportion of bee venom allergic subjects.

Nittner-Marszalska et al. recently described a surprisingly high incidence of VIT-related anaphylactic reactions in bee venom allergic adults (21.4%) which significantly exceeded the pediatric reaction rate of 7.2% (*P* = .034) [18]. No significant differences between pediatric and adult reaction rates were found by Birnbaum et al. [17].

The target venom dose of 100 µg was reached and tolerated in all our pediatric patients. The authors of two recent studies demonstrate a reasonable efficacy of 50 µg maintenance doses in children and therefore advocate the use of reduced doses in order to improve the safety while decreasing the costs of treatment [12, 13]. Given the good tolerability of 100 µg doses in children and the clear dose-dependency of VIT [31], we and others opt for the use of standard dose increase protocols with a target dose of 100 µg in pediatric patients [15, 16, 18].

## Conclusions

Our data demonstrate that, in contrast to previous assumptions [8], VIT-related pediatric anaphylactic reaction rates, are at least as high as those in adults. There is, however, need to clarify that VIT-induced anaphylactic reactions in children in both our series and previous studies [15–18] were invariably classified as mild to moderate and responded well to anti-allergic treatment, even without administration of epinephrine.

## Abbreviations

IQR: interquartile range
VIT: venom immunotherapy
